# Secondary Hemophagocytic Lymphohistiocytosis With Epstein–Barr Virus-Associated Transformed Follicular Lymphoma: A Case Report and Literature Review

**DOI:** 10.3389/fonc.2021.681432

**Published:** 2021-06-25

**Authors:** Huan Xu, Xia Xu, Guohui Cui, Jun Fang, Wanxin Chen, Mei Xue, Runming Jin, Hongbo Chen, Lu Zhang, Yu Hu

**Affiliations:** ^1^ Department of Pediatrics, Union Hospital, Tongji Medical College, Huazhong University of Science and Technology, Wuhan, China; ^2^ Department of Pathology, Union Hospital, Tongji Medical College, Huazhong University of Science and Technology, Wuhan, China; ^3^ Department of Hematology, Union Hospital, Tongji Medical College, Huazhong University of Science and Technology, Wuhan, China

**Keywords:** follicular lymphoma, histological transformation, Epstein–Barr virus, hemophagocytic lymphohistiocytosis, bone marrow involvement

## Abstract

A 58-year-old male was admitted to our hospital due to lasting fever, progressive lymphadenopathy and bicytopenia, with a previously histological diagnosis of follicular lymphoma grade 3a with Epstein–Barr virus-encoded RNA positive one month ago. A second biopsy of axillary lymph node revealed concurrent diffuse large B-cell lymphoma with Epstein–Barr virus-encoded RNA positive. Another diagnosis of hemophagocytic lymphohistiocytosis secondary to Epstein–Barr virus positive diffuse large B-cell lymphoma was further concluded by clinical manifestation, laboratory test and atypical lymphocytes in peripheral-blood smear. After a pulse of steroid pre-phase treatment, the patient’s clinical condition deteriorated and died in two weeks. The presence of Epstein–Barr virus infection in patients with follicular lymphoma is associated with more aggressive clinical course and increased risk of high-grade transformation. Hemophagocytic lymphohistiocytosis in response to Epstein–Barr virus infection or lymphoma remains fatal. Early diagnosis and initiation of treatment may improve the outcome.

## Introduction

Epstein–Barr virus (EBV) is a ubiquitous virus that latently affects nearly 90% population worldwide (1). It is first identified as an oncogenic virus in a Burkitt’s lymphoma cell line and subsequently found to be associated with the development of EBV-associated lymphoproliferative disorders, hemophagocytic lymphohistiocytosis (HLH), and solid tumors, among others. The oncogenic potential of EBV has been demonstrated by *in vitro* infection and the transformation of quiescent B cells into lymphoblastoid cell lines (LCLs) (2). It has been reported that the significant mechanism of EBV-induced lymphomagenesis relies on the capacity of the virus to establish a lifelong latent infection in B-lymphocytes. The expression of the highly immunogenic proteins is suppressed during latency of EBV, while viral lytic proteins are increased which impairs antigen processing by infected cells, and destroys the cellular molecular signaling machinery and metabolism to ultimately escape immune surveillance and promote tumor cell growth and survival.

Follicular lymphoma (FL) is the most common indolent and second most common non-Hodgkin lymphoma subtype. EBV-positive follicular lymphoma is a poorly characterized disease and is only rarely reported. It was described to share some similarities with EBV-positive diffuse large B cell lymphoma (DLBCL), especially in EBV positivity predominantly within large lymphoma cells, presence of EBV in latency II or III, lack of morphological or immunophenotypic features that predict EBV positivity, and lack of history of immunosuppression (3). Besides, EBV-positive FL is associated with grade 3 FL and the tendency of progression to higher grade FL or to DLBCL (3). These findings indicate the possibility that at least a subset of EBV-positive DLBCL may derive from EBV-positive FL. Although Asians generally have a higher incidence of EBV-associated malignancies as compared to western countries, rare findings on EBV-positive FL have been reported in the Chinese population.

EBV is also the most common infection highly associated with HLH, which is a rare syndrome of severe, life-threatening hyperinflammation. The underlying pathophysiology of HLH is the impaired activity of cytotoxic T lymphocytes and natural killer (NK) cells, leading to uncontrolled immune activation, hypercytokinemia, and macrophage proliferation. EBV-associated HLH is thought to be particularly prevalent in Asia with a fatal rate of up to 50% (4). This may be observed proceeding, concurrent with, or subsequent to EBV positive lymphoproliferative disorders (4). The majority of HLH-associated malignancies are T-cell and NK cell lymphomas. B-cell lymphoma-associated HLH has mainly been reported in the Asian population.

Here, we reported a very rare case of a patient with EBV-positive DLBCL accompanied with HLH may involve from EBV-positive FL within one month. This is a rare case of EBV-driven FL histological transformation as well as HLH in an immunocompetent patient.

## Case Presentation

A previously healthy 58-year-old man presented with a 2-month history of generalized lymphadenopathy, fatigue, night sweat, and weight loss. The Computed Tomography (CT) of the upper abdomen showed splenomegaly, multiple lymph nodes in the retroperitoneum and hilar region. An excisional biopsy of the left cervical lymph node was performed and histopathologic examination reported high-grade B cell lymphoma. The bone marrow involvement was excluded by flow cytometry and histology. The patient has no human immunodeficiency virus (HIV) viremia or evidence of immunosuppression.

**Figure 1:**
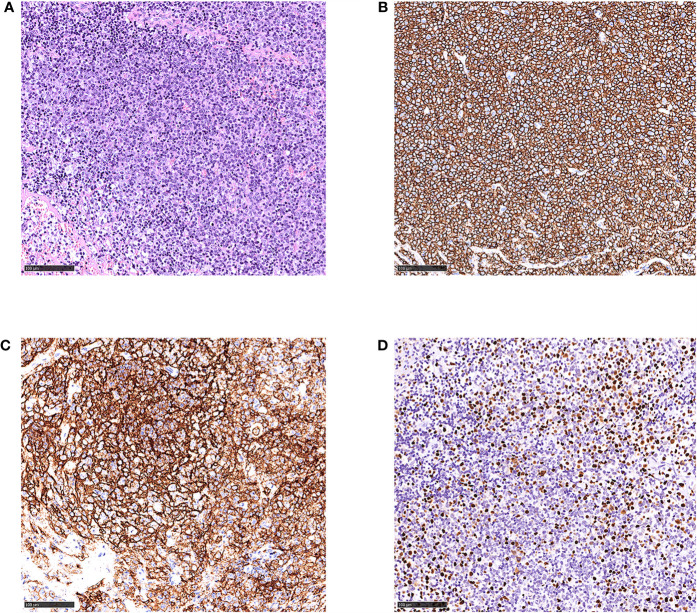
Histological and immunophenotypic results of excisional biopsy of left cervical lymph node. Hematoxylin and eosin (H&E)-stained sections is seen **(A)**. Immunohistochemical staining of these specimens shows that the infiltrated lymphocytes are positive for CD20 **(B)**, CD21 **(C)** of follicular dendritic cells, and EBER **(D)**. (magnification ×20).

**Figure 2:**
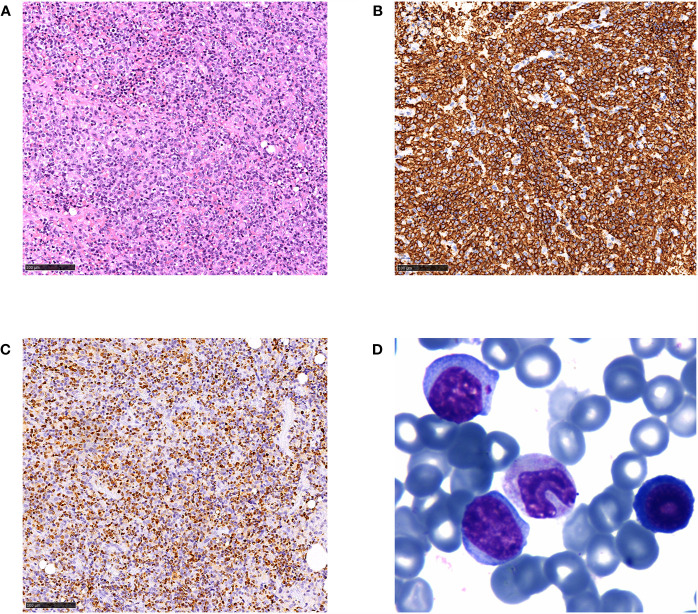
Histological and immunophenotypic results of excisional biopsy of the axillary lymph node and peripheral blood smear. H&E-stained sections is seen **(A)**, and immunohistochemical staining of the specimens shows that the diffuse infiltrated lymphocytes are positive for CD20 **(B)** and EBER **(C)** (magnification ×20). Wright’s staining of peripheral blood smear showed typical atypical lymphocytes **(D)** (magnification ×400).

One month later, the patient was transferred to our hospital due to a 10-days persistent fever with a maximum body temperature of 41°C, accompany by rapidly developing superficial lymphadenopathy. The results of pathological consultation in our hospital for the excisional biopsy of the left cervical lymph node reported a follicular lymphoma, grade 3a with EBER positive (**Figure 1**). Flow cytometry identified a population of kappa restricted B cells that were positive for CD19, CD20, CD79a, CD30 and negative for CD5, CD10, CD34, CD38, and Bcl-2. Large B cell transformation was suspected. Re-biopsy of the axillary lymph node supported the diagnosis of EBV-positive DLBCL, and atypical lymphocytes were noted in the peripheral blood smear (**Figure 2**). Bone marrow examinations indicated large B-cell lymphoma involvement without the sign of hemophagocytosis. While plasma cell-free EBV DNA (5.42 × 10^2^ copies/ml) and cellular EBV DNA (3.81 × 10^4^ copies/ml) were detected by quantitative PCR. A complete work-up was done including routine blood tests which showed leukocytosis, anemia, thrombocytopenia, coagulopathy, hypertriglyceridemia, hypoalbuminemia, hyperferritinemia, and elevated lactate dehydrogenase, and so on (**Table 1**). Further study included increased soluble CD25 (IL-2Rα) of 984.15 pg/ml (reference range: 3.71–16.05pg/ml), together with decreased NK cell function.

**Table 1:** Laboratory Examinations.

Variables	Results	Reference Range in Adults
Hemoglobin (g/L)	81	130-175
White blood cell count (G/L)	21.02	3.5-9.5
Platelet count (G/L)	100	125-350
Alkaline phosphatase (U/L)	642	38-126
Albumin (g/L)	25	35-50
Globulin (g/L)	24	23-32
Lactate dehydrogenase (U/L)	1732	109-245
Ferritin (μg/L)	5816.4	21.8-275
D-dimer (mg/L FE)	4.34	<0.5
Fibrinogen FIB (g/L)	1.07	2.0-4.0
Erythrocyte sedimentation rate ESR (mm/h)	88	<15
β2-microglobulin (mg/L)	6.5	1.0-3.0
Hypersensitive C-reactive protein (mg/L)	85.1	<8.00
Immunoglobulin A (g/L)	0.56	0.82-4.53
Immunoglobulin M (g/L)	3.67	0.46-3.04
Complement C3 (g/L)	0.414	0.79-1.52
Complement C4 (g/L)	0.114	0.16-0.38
HIV Ag/Ab (S/CO)	Negative	Negative
EBV-DNA intracellular (copies/ml)	38100	<400
EBV-DNA extracellular (copies/ml)	542	<400
Cytomegalovirus CMV-DNA (copies/ml)	<400	<400
Triglyceride (mmol/L)	2.27	<1.7

A total-body CT scan confirmed systemic lymphadenopathy, together with hepatosplenomegaly. To complete the staging procedures, the 18F-FDG PET/CT was performed showing generalized lymphadenopathy with FDG avidity, and diffuse FDG uptake of the spleen and bone marrow. Based on the clinical and laboratory manifestation, along with the re-biopsy results, a final diagnosis of HLH secondary to EBV-positive DLBCL was made. The patient’s clinical condition rapidly deteriorated and he died of multiple organ dysfunction shortly after receiving a pulse of steroid pre-phase treatment (dexamethasone, 15 mg, qd).

## Discussion

EBV-associated follicular lymphoma is poorly characterized and only rarely reported. A recent retrospective study of 382 follicular lymphoma patients suggested that the prevalence of EBV infection in the FL is about 2.6% in Western populations (3). In this case series, EBV-positive FL was characterized by grade 3A-3B FL, CD30 expression, rapid progression of the disease, and inferior overall survival. Moreover, the majority of EBV-positive FL cases had progressed to higher grade FL or EBV-positive DLBCL (3). Currently, the relationship between EBV and FL occurring in the setting of immunocompetence is not well recognized. Only a few sporadic cases of EBV-positive FL have been reported in the literature. Koreishi et al. firstly reported a case of a 65-year-old woman who had the simultaneous occurrence of FL, Kaposi sarcoma (KS), and Castleman’s disease (CD) with a coinfection of EBV and HHV-8 in 2011. The FL component was grade 3A and large neoplastic centroblasts were positive for EBER (5). The patient was likely immunosuppressive, thus this case is distinct from our patient. Then Menon et al. reported a 53-year-old white woman who was diagnosed as grades 1–2 FL and was EBV negative in 2013. However, the patient progressed to grade 3A FL with classic Reed-Sternberg (RS) and Hodgkin cells-like morphology after a year of treatment. Moreover, EBER was positive in Hodgkin-like cells as well as in fewer background cells in the neoplastic follicles (6). Orlandi et al. also reported a case of grades 1–2 FL in a 30-year-old male which transformed to a fatal EBV associated DLBCL after treatment with multiple chemotherapy regimens, which possibly due to the iatrogenic immunosuppression and uncontrolled reactivation of latent EBV (7).

**Figure 3:**
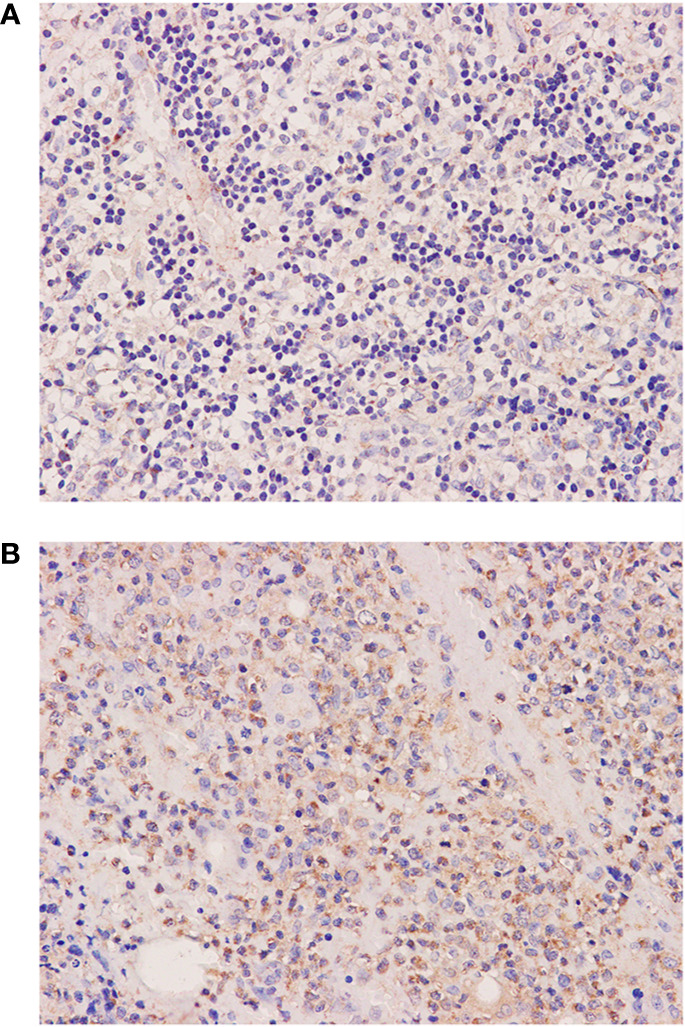
Histological and immunophenotypic results of two excisional biopsies of lymph nodes. Immunohistochemical staining of excisional biopsy of the left cervical lymph node shows the expression of AID **(A)**. Immunohistochemical staining of excisional biopsy of the axillary lymph node shows an increase of AID expression in large transformed cells **(B)**. (magnification ×400).

In our case, the initial diagnosis was grade 3A FL with EBER and CD30 expression and without evidence of immunodeficiency. Within a month, this patient followed an unusually aggressive clinical course with lasting high fever, rapidly enlarging lymphadenopathy, and cytopenia. Another biopsy is in urgent need and the result led to another diagnosis of EBV-positive DLBCL, while cell-free EBV-DNA raised to 5.42 × 10^2^ copies/ml from zero and lymphoma cells spread quickly to the bone marrow. Elevated levels of EBV DNA in the blood of transplant recipients were reported to be associated with an increased risk of developing post transplanted lymphoproliferative disorders (PTLD). Some studies have certified that EBV infection was implicated as an etiologic risk in the Ritcher’s transformation of CLL to Hodgkin-like lymphoma, and the presence of EBV-EBER-1 and EBV-encoded latent membrane protein-1 (LMP1) mRNA in patients with CLL was associated with poor outcomes (8–10). Mackrides et al. indicated that EBV is present in latency type II or III in EBV-positive FL. They also concluded that EBV-positive FL was more frequently associated with grade 3A-3B and significantly related with CD30 expression (3). It is rational to hypothesize that the EBV infection may play a role in lymphomagenesis and transformation in a subset of FL. It is also noteworthy that activation-induced cytidine deaminase (AID) is overexpressed on the large transformed cells in our case (**Figure 3**). EBV-LMP1 is a transmembrane protein that is essential for EBV-induced immortalization and transformation of B cells. AID is an enzyme that is dominantly expressed in germinal center B cells and plays an essential role in somatic hypermutation and class switch recombination of immunoglobulin genes in the normal B-lymphocyte development (11). Deregulated AID expression acts as a genomic mutator, leading to the development of B-cell lymphoma (12). In addition, infection of human B cells with EBV was observed to result in AID expression (13). Kim et al. also demonstrated that LMP1 increased AID mRNA expression and promoter activity, increased AID expression, in turn, promoted genomic instability and down-regulation of the NF-κB inhibitor, Rassf6, thereby further increasing the survival of genetically destabilized B-cell lymphoma cells (14). Ma et al. also indicated that EBV-encoded microRNAs can regulate the expression of cellular messenger RNAs and the interaction have significant effects in the regulation of cellular pathways, such as cell survival, apoptosis, and proliferation pathways (15). These findings suggest that AID may play a role in EBV-induced lymphomagenesis, high-grade transformation, and increased aggressiveness in B cells. Further studies should focus on the expression of AID in EBV-positive FL to evaluate the relationship with prognosis. We know that the vast majority of FL occur without EBV infection, therefore we believe that EBV infection is a facilitator of FL progression instead of the driver event of EBV-positive FL in most cases. To better understand the contribution of EBV infection to lymphomagenesis and FL progression, gene expression analysis of sorted FL cells (EBV-positive *versus* EBV-negative) in the same patient may be a useful method.

A clinical diagnosis of HLH was suspected when our patient developing symptoms of hepatosplenomegaly, cytopenia besides persistent fever, while the EBV DNA in the plasma was significantly high. This diagnosis was further confirmed by laboratory examinations according to revised diagnostic criteria of HLH-2004 (16). The main pathophysiology of HLH is the impaired activity of cytotoxic T lymphocytes and NK cells, leading to uncontrolled immune activation, hypercytokinemia, macrophage proliferation, and immune-mediated injury of multiple organ systems (17). HLH comprises primary (genetic) and secondary (infection, malignancy, and autoimmune disease-associated) forms. Viral infection is the most frequent immunologic trigger (18). Approximately half of all infection-associated HLH cases involve EBV (19). Among the malignancies, hematological malignancies are more frequent, especially lymphomas. EBV infection was reported to play an important role in lymphoma-associated hemophagocytic syndrome (LAHS), and the prevalence of which as a co-trigger varies between up to 90% in Hodgkin’s lymphoma, to about 33% in peripheral T-cell lymphoma, while it is very rare in DLBCL (20). The EBV-related HLH might be seen preceding, concurrent with, or subsequent to systemic EBV lymphoproliferative disease. In our case, the patient developed HLH during the transformation of EBV-positive FL to DLBCL, thus both the EBV virus and lymphoma drove the occurrence of HLH.

Bone marrow involvement of lymphoma appears to be another risk factor for developing HLH in our case. Prior studies had noticed that bone marrow involvement is related to increased risk of developing HLH in patients with T-cell lymphoma and extranodal NK/T-cell lymphoma (21). Yeh et al. had described an aggressive entity of large B cell lymphoma, which presenting initially in the bone marrow, liver, and spleen, was frequently associated with HLH (22). However, the mechanism of bone marrow involvement contributing to the pathogenesis of HLH remains unclear, and we need more clinical data and studies to verify the relationship between bone marrow involvement of lymphoma and HLH.

The management of HLH remains challenging. Once EBV-positive DLBCL was diagnosed, a pre-phase treatment comprising steroid was initiated to reduce tumor burden and facilitate the clearance of viruses residing in these cell populations. However, the patient died of multiple organ failures in two weeks. Currently, in addition to HLH-directed therapies, treatment of underlying triggers is recommended. The anti-CD20 antibody rituximab can deplete EBV-harboring B cells and improve the outcome of EBV-associated HLH (23). Chellapandian et al. reported that combined rituximab with the conventional HLH therapies resulted in a significantly reduction in EBV load and was associated with significant decreasing in ferritin levels (24). For our patient, before initiating anthracycline-based chemotherapy, adding rituximab to the steroid pre-phase might be more appropriate to reduce lymphoma burden and EBV load.

These findings remind us of the significance of the identification of EBV infection, histological transformation, as well as bone marrow involvement in patients with high-grade follicular lymphoma who experience a very aggressive clinical course. Further studies are required to solve these questions regarding whether there is a prognostic or therapeutic significance of identifying EBER, AID, and CD30 expression in FL, and whether identifying if an EBV-positive DLBCL has derived from another EBV-positive lymphoma especially FL has clinical significance. The presence of Epstein–Barr virus infection in patients with follicular lymphoma is associated with a more aggressive clinical course and increased risk of high-grade transformation. Hemophagocytic lymphohistiocytosis in response to Epstein–Barr virus infection or lymphoma remains fatal (25). Early diagnosis of HLH and initiation of rituximab-contained regimens may improve their outcomes.

## Data Availability Statement

The original contributions presented in the study are included in the article/supplementary material. Further inquiries can be directed to the corresponding authors.

## Ethics Statement

The studies involving human participants were reviewed and approved by the Medical Ethics Committee of Union Hospital, Tongji Medical College, Huazhong University of Science and Technology. The patients/participants provided their written informed consent to participate in this study.

## Author Contributions

HX composed the manuscript and literature review. XX, WC and MX provided figures and pathology interpretation. GC, JF, RJ, HC, LZ and YH had the acquisition, analysis or interpretation of data for the work, and final approval of the version to be published. All authors contributed to the article and approved the submitted version.

## Funding

The project was supported by the National Natural Science Foundation of China (No.81400172).

## Conflict of Interest

The authors declare that the research was conducted in the absence of any commercial or financial relationships that could be construed as a potential conflict of interest.
